# The role of the corticotropin-releasing hormone and its receptors in the regulation of stress response

**DOI:** 10.18699/VJ21.025

**Published:** 2021-03

**Authors:** E.V. Sukhareva

**Affiliations:** Institute of Cytology and Genetics of the Siberian Branch of the Russian Academy of Sciences, Novosibirsk, Russia

**Keywords:** corticotropin-releasing factor, corticotropin-releasing factor receptors, stress, psychopathology, hypothalamus, extrahypothalamic brain structures., кортикотропин-рилизинг фактор, рецепторы кортикотропин-рилизинг фактора, стресс, психопатологии, гипоталамус, внегипоталамические структуры головного мозга

## Abstract

Stress is an essential part of everyday life. The neuropeptide corticotropin-releasing hormone (CRH, also called CRF and corticoliberin) plays a key role in the integration of neuroendocrine, autonomic and behavioral responses to stress. The activation of the hypothalamic-pituitary-adrenal axis (HPA axis) by neurons of the paraventricular hypothalamic nucleus (PVN), the primary site of synthesis CRH, triggers stress reactions. In addition to the hypothalamus, CRH is widespread in extrahypothalamic brain structures, where it functions as a neuromodulator for coordination and interaction between the humoral and behavioral aspects of a stress response. The axons of neurons expressing CRH are directed to various structures of the brain, where the neuropeptide interacts with specific receptors (CRHR1, CRHR2) and can affect various mediator systems that work together to transmit signals to different brain regions to cause many reactions to stress. Moreover, the effect of stress on brain functions varies from behavioral adaptation to increased survival and increased risk of developing mental disorders. Disturbances of the CRH system regulation are directly related to such disorders: mental pathologies (depression, anxiety, addictions), deviations of neuroendocrinological functions, inflammation, as well as the onset and development of neurodegenerative diseases such as Alzheimer’s disease. In addition, the role of CRH as a regulator of the neurons structure in the areas of the developing and mature brain has been established. To date, studies have been conducted in which CRHR1 is a target for antidepressants, which are, in fact, antagonists of this receptor. In this regard, the study of the participation of the CRH system and its receptors in negative effects on hormone-dependent systems, as well as the possibility of preventing them, is a promising task of modern physiological genetics. In this review, attention will be paid to the role of CRH in the regulation of response to stress, as well as to the involvement of extrahypothalamic CRH in pathophysiology and the correction of mental disorders.

## Abstract (ru)

Стресс является неотъемлемой частью повседневной жизни. Нейропептид кортикотропинрилизинг гормон (CRH, также называемый CRF и кортиколиберином) играет ключевую роль в интеграции нейроэндокринных, вегетативных и поведенческих реакций на стресс. Активация гипоталамо-гипофизарной-надпочечниковой системы нейронами паравентрикулярного ядра гипоталамуса (PVN), основного места синтеза CRH, запускает стрессорную реакцию. Помимо гипоталамуса, CRH широко распространен во внегипоталамических структурах головного мозга, где он функционирует как нейромодулятор для координации и взаимодействия между гуморальными и поведенческими аспектами реакции на стресс. Аксоны нейронов, экспрессирующих CRH, направляются к различным структурам головного мозга, где нейропептид взаимодействует со специфическими рецепторами (CRHR1, CRHR2) и может оказывать влияние на различные медиаторные системы, которые действуют сообща для передачи сигналов в разные области мозга, чтобы вызвать множество реакций на стресс. При этом воздействие стресса на функции мозга варьирует от поведенческой адаптации до повышения выживаемости и увеличения риска развития психических расстройств. Нарушения в регуляции системы CRH непосредственно связаны со следующими расстройствами: психическими патологиями (депрессией, тревожностью, зависимостями), отклонениями нейроэндокринологических функций, воспалением, а также с возникновением и развитием нейродегенеративных заболеваний, таких как болезнь Альцгеймера. Кроме того, установлена роль CRH как регулятора структуры нейронов в областях развивающегося и зрелого мозга. На данный момент проведены исследования, в которых CRHR1 выступает мишенью для антидепрессантов, в качестве которых используются антагонисты этого рецептора. В связи с этим исследование участия системы CRH и его рецепторов в негативных эффектах на гормон-зависимые системы, а также возможности их предотвращения является перспективной задачей современной физиологической генетики. В настоящем обзоре внимание уделено роли CRH в регуляции реакции на стресс, а также участию внегипоталамического CRH в патофизиологии и коррекции психических расстройств.

## Corticotropin-releasing hormone system

The mammalian family of CRH peptides includes CRH, urocortin 1 (UCN1), urocortin 2 (UCN2), and urocortin 3 (UCN3) (Hauger et al., 2006). These 38–41 amino acid peptides are structurally related and have high (26–54 %) sequence identity (Dautzenberg, Hauger, 2002). These peptide hormones and their receptors are ubiquitous in mammalian tissues, playing a key role in stress-mediated effects. Although CRH was originally described as a regulator of the hypothalamic-pituitary-adrenal system (HPA), the peptide is widely expressed in brain regions as well as in peripheral tissues, including the heart, blood vessels, skin, lungs, spleen, pancreas, kidneys, liver, adipose tissue, gastrointestinal tract, testes, ovaries and placenta (Hauger et al., 2006). Urocortins expression in the brain is more limited than CRH expression and is found predominantly in the Edinger–Westphal nucleus, as well as in the supraoptic nucleus, pituitary gland, lateral superior olive, cardiovascular system, skeletal muscle, kidney, adipose tissue, digestive tract and genital glands (Waters et al., 2015).

The Crh gene is located on the long arm of chromosome 8 (8q13) and consists of a promoter sequence, one intron, and two exons. In humans, sheep, mice, and rats, the Crh gene promoter sequence has 97 % homology over the first 270 bp. The gene encodes an inactive pro-hormone of 196 amino acids (pro-CRH) (King, Nicholson, 2007).

Corticoliberin and urocortins act through two receptors, CRHR1 and CRHR2, which belong to the Gs-protein-coupled receptor family. These receptors are encoded by different genes, but they have 70 % identity at the amino acid level, and their main divergence is found in the ligand-binding domains, and, therefore, are responsible for their selectivity towards agonists (Dautzenberg, Hauger, 2002). CRH is a high affinity ligand for CRHR1 and binds poorly to CRHR2, for which other related CRH peptides, UCN2 and UCN3, have higher affinity. UCN1 has the same affinity for both CRHRs (Hauger et al., 2006). The amino acid sequences of the intracellular and transmembrane domains of CRHR have 80–85 % identity. CRHRs belong to the class B G-protein-coupled receptors (secretin-receptor family) (Markovic, Grammatopoulos, 2009), which bind polypeptide hormones. As a common feature of the family, they exhibit a variety of splicing variants that can contribute to tissue-specific differences in ligand binding to the receptor. Several variants of CRHR1 splicing (CRHR1α and CRHR1β) have been identified, but only one, CRHR1α, has biological activity, and its expression predominates (Zmijewski, Slominski, 2010). In humans, CRHR2 exists in three variants of CRHR2α splicing, β and γ (only two isoforms are found in mice), which differ in amino-terminal domain and tissue distribution. CRHR2α is the most common splicing variant in the brain; CRHR2β is found exclusively in peripheral tissues such as the retina, gonads, digestive tract, heart, skeletal muscle, lungs, and skin, while CRHR2γ is found in the septum, hippocampus, amygdala, midbrain, frontal cortex, and limbic regions of the human brain, however function not defined (Hauger et al., 2006). In terms of the expression level and its distribution, CRHR1 is the main CRH receptor in the brain, while the representation of CRHR2 is limited (Henckens et al., 2016).

The CRH system also includes a corticotropin-releasing hormone binding protein (CRH-BP), which is expressed in peripheral tissues and the brain. Previously it was thought that it binds free CRH and CRH-associated ligands, which attenuates receptor activation, but now its physiological role seems more complex and is still being studied. For example, in the ventral tegmental area (VTA), the effects of CRHR2 activation are highly dependent on CRH-BP (Wang B. et al., 2007), and recent in vitro data suggest a role for CRH-BP in CRHR2 localization at the cell membrane (Slater et al., 2016).

## Action mechanism of corticotropin-releasing hormone

Activated CRHR1 and CRHR2 primarily transmit signals through protein binding, which leads to the induction of the cAMP/PKA molecular cascade (Deussing, Chen, 2018). PKA phosphorylates a variety of substrate proteins, including CREB, which regulates the expression of various target genes in the nucleus. In parallel, cAMP activates the EPAC factor (exchange protein directly activated by cAMP), which, in turn, triggers the MAP kinase cascade, which ultimately leads to phosphorylation of ERK1/2 (Van Kolen et al., 2010). ERK1/2 activates various transcription factors including Nur77. However, both CRH receptors can activate any Gα-subunit, albeit with different activities: Gs≥Go>Gq/11>Gi1/2>Gz (Deussing, Chen, 2018), thereby activating the phospholipase C (PLC) cascade and leading to activation of ERK1/2 and an increase in the intracellular concentration of Ca2+ (Grammatopoulos, Chrousos, 2002). Gs-protein binding also leads to intracellular Ca2+ mobilization (Gutknecht et al., 2009). At the same time, the binding of receptors to the Gi -protein blocks the activity of adenylate cyclase (Deussing, Chen, 2018). Thus, depending on their localization and cellular context, CRH receptors can have multiple effects (Deussing, Chen, 2018).

After CRHR activation G-protein-coupled receptor kinases (GRKs), mainly, but also protein kinases A or C (PKA and PKC), rapidly phosphorylate receptors, desensitize and increase their affinity for β-arrestins (Deussing, Chen, 2018). These processes can also alter the receptor signaling pathways. β-arrestins act as adapter proteins that bind to proteins involved in receptor endocytosis, clathrin and β-adaptin, to initiate the internalization of CRHR1 and CRHR2 via clathrincoated vesicles (Markovic et al., 2008). The receptors are then either dephosphorylated, resensitized, and reinserted into the plasma membrane, or (upon prolonged exposure to high concentrations of the agonist) are degraded in lysosomes, which leads to a decrease in the number of receptors (Kelly et al., 2008). Severe exposure to stress can influence these processes and contribute to the degradation of CRH receptors (Reyes et al., 2008). It is important to note that not all phosphorylated receptors are transferred to endosomes (internalized); some remain on the membrane (Deussing, Chen, 2018).

CRH binding to its receptors activates the ACTH release from pituitary corticotropic neurons (Herman, Tasker, 2016). ACTH stimulates the synthesis and release of glucocorticoids from the adrenal cortex, cortisol in primates and corticosterone in rodents. The biological effects of glucocorticoids support adaptation to stress-induced needs by controlling energy metabolism (Herman et al., 2003). The adaptive function of the HPA depends on negative feedback mechanisms that bring the system to basal levels (Quax et al., 2013). It should be noted that the Crh gene promoter does not contain the classical glucocorticoid response element (GRE), but there is evidence that glucocorticoids can regulate the Crh expression through protein-protein interactions (Nicholson et al., 2004; Kalinina et al., 2016). An atypical GRE/AP-1 element is located in the –278/–249 region of the human CRH gene promoter, where a specific, high-affinity glucocorticoid receptor binding was found, which provides dexamethasone-dependent inhibition of CRH gene transcription through protein-protein interactions. Removal of this region reduced glucocorticoid-dependent repression of the CRH gene promoter activity (Aguilera, Liu, 2012). Also, glucocorticoids have negative regulation of cAMP-stimulated activity of the CRH gene promoter, but not basal activity, which suggests that the glucocorticoids effect depends on the interaction with the CRE-associated transcription complex.

## CRH and its receptors expression, participation in the stress response

CRH plays an important role in the regulation of the HPA, the main stress response system. CRH is synthesized in the neurons of the hypothalamic paraventricular nucleus (PVN), which are innervated from both the limbic and brainstem centers, allowing them to respond to both physical and psychological stressors (Lightman, 2008). Three main groups of neurons were described in this core:

- Anterior and medial-dorsal parvocellular CRH neurons, whose axons are directed to the portal vessels of the external zone of the median eminence. Neurons in this area are classified as parvocellular due to their small size compared to large neurons. CRH neurons in this region co-express arginine-vasopressin (AVP), enkephalins, cholecystokines, and angiotensin I;
- Dorsolateral magnocellular vasopressinergic and oxytocinergic neurons, whose axons are directed to the posterior pituitary gland through the inner zone of the median eminence. These neurons release peptides into the peripheral circulation. Oxytocinergic neurons, but not vasopressinergic ones, also express CRH and respond to osmotic and non-osmotic stressors;
- Autonomic CRH neurons in the dorsal, medial-ventral and lateral parts of the PVN, with projections onto the brainstem and spinal cord. Neurons in this area express CRH and other neuropeptides and involved in the regulation and sympathoadrenal system (Aguilera, Liu, 2012)

In addition to PVN, CRH mRNA and protein are found in other brain areas, including limbic and other structures associated with stress responses, such as the nucleus of the end streak bed (BNST), the central nucleus of the amygdala (CeA), the locus coeruleus of the brainstem (LC), cerebral cortex, hippocampus, cerebellum, Barrington’s nucleus (Bar), spinal cord segments with projections from the midbrain and pons (Kono et al., 2016). However, the functions of CRH-secreting neurons differ significantly depending on the region in which they are located. For example, neurons of the inferior olive (IO) expressing CRH send axons to the cerebellum and play an important role in the induction of synaptic plasticity of the dendritic synapses of Purkinje cells (Andres et al., 2013). IO electrical stimulation increases the amount of CRH in the cerebellum, demonstrating the physiological effect of endogenous CRH in the cerebellum (Tian, Bishop, 2003). CRH neurons in the cerebral cortex are interneurons and can modulate the activity of pyramidal neurons (Gallopin et al., 2006). In the BNST, CRH neurons are involved in recovery induced by stress (Erb, Stewart, 1999) and alcoholism (Pleil et al., 2015). At an early age, a short-term increase in CRH expression in the forebrain causes long-term anxiogenic and desperate behavioral changes in mice (Kolber et al., 2010). In CeA CRH may also be associated with stress-induced anxiety (Regev et al., 2012), and in the basolateral amygdala CRH may alter the process of memory consolidation under stress (Roozendaal et al., 2002). In Bar CRH neurons send projections to the lumbosacral spinal cord and directly (Studeny, Vizzard, 2005) or indirectly (Sasaki, Sato, 2013) control the urinary reflex.

CRH is also present in other nerve structures, such as chromaff in cells in the adrenal medulla and sympathetic ganglia of the autonomic nervous system, as well as in non-neuronal peripheral organs such as the ovaries, testes, intestines, heart, lungs, and spleen (Stengel, Taché, 2010). Within the spleen, Crh mRNA is localized specifically in T-lymphocytes.

Glucocorticoids are the main element in the regulation of CRH expression in the hypothalamus by the type of negative feedback, which is mediated by the hormones activation by glucocorticoid receptors (GRs). GRs are widely expressed in the medial parvocellular part of the PVN and are co-localized with CRH, which promotes rapid inhibition of CRH neurons through non-genomic signaling. This mechanism is the key to limiting the duration of glucocorticoid secretion after acute stress, since genomic feedback will not be fast enough for timely termination of HPA system activation (Herman, Tasker, 2016). Dexamethasone (a synthetic glucocorticoid) reduces the cAMP-stimulated CRH level by more than 50 % in AtT20 cells (a mouse pituitary tumor cell line that produces proopiomelanocortin (POMC)) (Abou-Seif et al., 2012). Negative feedback from glucocorticoids is a critical characteristic of HPA system, which is lost in ACTH-secreting adenomas (Perez-Castro et al., 2012) and in some patients with depression (Reul, Holsboer, 2002), creating an excess of glucocorticoids. GRs leads to both inhibition of ACTH release and repression of Pomc transcription. Removal of the Crh gene blocks both basal and stress-induced release of ACTH, which indicates the obligatory CRH involvement in the HPA activation (Muglia et al., 2000). The chronic effect of noise as a stressor increases the Crh mRNA level in the hypothalamus, while reducing the Gr mRNA level in the hippocampus. Both acute and chronic effects of noise increase the Crhr1 mRNA level in the hypothalamus, but decrease it in the hippocampus. These data show that the involvement of CRH and GR in noise stress responses is different and depends on the brain area (Eraslan et al., 2015).

Disturbances in the parvocellular area of the PVN reduce the manifestation of anxiety in a new environment. Optogenetic inhibition of CRH expression in PVN neurons reduces stressinduced grooming and improve spatial orientation and learning after stress, while stimulation induces grooming and reduces exploratory behavior (Fuzesi et al., 2016). These data confirm that CRH PVN neurons may be involved in the coordination of behavioral as well as neuroendocrine responses to stress.

## Extrahypothalamic CRH: organizing and integrating stress response

CRH controls the stress response by acting on the LC neurons, the adrenal medulla, and the peripheral sympathetic nervous system. CRH plays a key role in coordinating the peripheral stress response systems and the central norepinephrine release in response to stress. It is believed that the influence of CRH on norepinephrine release, mainly in the LC, underlies the emotional basis of the stress response (Valentino, Van Bockstaele, 2008).

Glucocorticoids, as mentioned earlier, through negative feedback inhibit the release of CRH and ACTH in the hypothalamus and pituitary gland, respectively. However, in extrahypothalamic brain structures, the glucocorticoids action stimulates rather than inhibits CRH synthesis (Kovacs, 2013). Pathologies such as anxiety and affective disorders caused by an increased glucocorticoids level and disorders in the functioning of the brain neurotransmitter systems may be due to an increased CRH expression in CeA and BNST (Donner et al., 2016). Thus, the dexamethasone introduction in the neonatal period of development leads to an increase in the Crh expression in the brainstem in the first hours after the injection and to a complete restoration of the initial expression level by 6 hours, while the tyrosine hydroxylase gene expression (Th, a key enzyme in the norepinephrine synthesis) in this the same brain structure increased by 6 hours after dexamethasone administration, and the effect persisted 24 hours after exposure. However, preliminary administration of the CRHR antagonists – antalarmine and antisauvagin-30 before glucocorticoid therapy suppresses the increase of Th expression in the brainstem of neonatal rat pups caused by a single dexamethasone injection (Sukhareva et al., 2019). Based on this, it can be assumed that the use of these drugs can prevent the negative effects of hormone therapy in newborns in adulthood.

Unlike classical neurotransmitters, CRH and its related peptides act as neuroregulators: without affecting synaptic efficiency, they activate signaling processes in cells that facilitate or suppress the neurotransmitters action in certain neural networks. The CRH system is anatomically and functionally associated with monoaminergic systems, which, acting together, transmit stress signals by altering the biosynthetic activity of neurons in different brain regions, thereby inducing different responses to stress (Gallagher et al., 2008).

CRH released by extrahypothalamic brain structures directly contributes to behavioral anxiety, regardless of its effect on the pituitary gland and the sympathetic system, since an anxiety-like effect after intraventricular CRH administration persists in hypophysectomized rats (Inda et al., 2017). In rodents, increased CRH expression in the brain induces an anxiogenic behavioral phenotype (Van Gaalen et al., 2002), while suppression of CRH expression has an anxiolytic effect in basal and stress-induced anxiety (Henckens et al., 2016). CRH levels are elevated in the brains of people suffering from stress-related mental illnesses such as major depressive disorder and post-traumatic stress disorder (PTSD) (Rasmusson, Pineles, 2018) and, in some cases, normalize after treatment with antidepressants (Inda et al., 2017).

The CRH anxiogenic effects are associated with the CRHR1 receptor activation. CRHR1 blockade in rodents prevented the CRH-induced anxiogenic phenotype (Zorrilla et al., 2002), and in mice lacking Crhr1, the manifestation of anxiety behavior decreased (Muller et al., 2003). These results have awakened interest in the role of CRHR1 hyperactivation in stress-related psychopathology, and also opened up the possibility of using CRHR1 antagonists as potential next-generation anxiolytics and antidepressants.

In contrast to CRHR1, the role of CRHR2 activation in the manifestation of anxiety and depression is less clear, and there are two theories trying to explain the CRHR2 involvement in the CRH behavioral effects. The most common hypothesis – CRHR2 activation is responsible for providing physiological and psychological homeostasis and counteracts the initial effects of CRHR1 activation, which induce stress response and anxiety-like behavior (Bale, Vale, 2004). This assumption is based on data obtained in Crhr2 knockout mice, which have an increased release of corticosterone under stress, an anxiogenic phenotype (Bale et al., 2000), and an increase in recovery time after stress (Issler et al., 2014). Similar manifestations were noted in mice that lacked all three urocortins (UCN1–UCN3), the primary ligands for CRHR2 (Neufeld-Cohen et al., 2010). An alternative hypothesis for the role of CRHR2 – CRHR1 and CRHR2 are responsible for opposite types of stress-related behavior. CRHR1 mediates active defensive behavior (caused by controlled stress), while CRHR2 mediates passive coping behavior and depression-like reactions (such as learned helplessness caused by uncontrolled stress) (Maier, Watkins, 2005). This hypothesis is based on the necessity of CRHR2 signaling in the dorsal raphe nuclei for sensitization of serotonergic neurons and the development of a depressive-like phenotype that occurs under conditions of unavoidable stress. However, there are a number of results that do not agree with these theories (Janssen, Kozicz, 2013). This contradiction may be due to the erroneous determination of the CRH and related peptides contribution and their receptors to the observed effects due to their partially overlapping distribution patterns, not the absolute specificity of receptor activation: high concentrations of the ligand act on both receptors types, and receptor antagonists do not have specificity (Zorrilla et al., 2013).

Different types of stressors require different physiological responses in order to deal with them optimally. Not only the nature, but also the intensity and duration of the stressor influences the required neuronal response (Joels, Baram, 2009). Since the CRH system acts immediately, exerting its neuromodulatory effect on target neurons within a few seconds after its release (Gallagher et al., 2008), it is worth noting the rapid effects of CRH receptor signaling, either in the presence or absence of chronic stress.

There is a lot of data indicating that in the case of long-term exposure, CRH has effects that are fundamentally different from acute ones (Maras, Baram, 2012), and these effects are associated with a depressive rather than anxiety phenotype (Regev et al., 2011). Long-term CRH exposure and CRHR1 activation impair the neuroplasticity processes in the hippocampus. The initially activating CRH effects in vitro with long-term exposure to CRH are replaced by a decrease in excitatory postsynaptic potentials, which blocks short- and long-term synaptic plasticity, as well as actin polymerization. CRH destabilizes and thus thins dendritic spines, which leads to a decrease of excitatory synapses which ready for potentiation (Chen et al., 2013). These effects are mediated by local CRHR1-induced activation of NMDA receptors.

In terms of behavior, long-term CRHR1 activation as a consequence of chronic stress is associated with impaired potentiation response in the hippocampus. Mice lacking CRHR1 in forebrain neurons show no negative effects of chronic stress on learning and memory (Wang X. et al., 2011), and CRHR1 antagonist administration to wild-type rodents immediately after exposure to a stressor restores long-term potentiation (LTP) and integrity dendritic structure (Ivy et al., 2010). Thus, in contrast to the positive effects of short-term exposure to basal CRH levels, the peptide exposure under severe stress, when the hormone level is increased, leads to the loss of synapses for a longer period (Chen et al., 2012). Consistent with this, memory impairments are common in people with post-traumatic stress disorder (PTSD) (Brewin et al., 2007), and repeated injection of the selective CRHR1 antagonist prevents this cognitive impairment and the associated decrease in the excitability of hippocampal neurons in a mouse model of PTSD (Philbert et al., 2013). Differences in the manifestations of short-term and long-term CRH exposure emphasize the importance of carefully studying the effects of CRHR signaling over a long period of time (hours, days) and the importance of the mechanisms behind their constancy over time. Factors such as age, gender, and genetics, often in conjunction with life events, are the main determinants of the effects observed after the CRH system activation (Koenig et al., 2011).

Differential expression of receptors and their ligands in the brain reflect the different actions that CRH has at the CNS level. The results show a more complex modulating role for the CRH system than the conventional wisdom, where CRHR1 causes anxiety and CRHR2 mediates recovery after stress. While CRH-mediated activation of CRHR1 increases basal and stress-induced anxiety in many brain areas, including BLA, BNST, and PAG, a more complex picture is observed for other brain regions. For example, in the globus pallidus, CRHR1 activation is associated with anxiolytic effects, whereas the potential anxiogenic effects of CRHR1 activation in CeA appear to depend on the strength and duration of stress (Henry et al., 2006). The same is observed in NAc. In addition, the effects of CRHR2 activation appear to be dynamic and highly dependent on the amount of endogenous ligand and prior exposure.

 It is known that repeated daily subthreshold activation of CRH receptors in the BNST induces a state similar to chronic anxiety. BNST neurons are largely projected onto neurons in the dorsal raphe nuclei (DR), the main source of serotonin in the brain. Repetitive CRHR activation in BNST, caused anxiety, increases the genes expression of the serotonergic system in DR, including Tph2 (a gene of a key serotonin synthesis enzyme) and Slc6a4 (a gene encoding a serotonin transporter (SERT)) (Donner et al., 2020). CRH overexpression in BNST neurons selectively decreases the CRHR2 expression within the dorsomedial DR and increases the manifestation of emotional memory (Sink et al., 2013). Conversely, serotonergic projections from DR to CRH neurons in BNST promote the development of anxiety through 5-HT2C receptors activation (Marcinkiewcz et al., 2016). Direct CRH neurons activation in the nucleus ovale of the BNST induces the anxiety that occurs after chronic stress. Thus, CRH neurons in the nucleus oval of the BNST and serotonergic neurons in DRD appear to have reciprocal connections that play a role in the control of emotional behavior, including defensive behavioral responses similar to anxiety.

## Conclusion

The effects of stress on brain function are diverse. These include behavioral adaptation, and increased survival, as well as an increased risk of developing mental disorders associated with stress. Based on the presented data, the activity of the hypothalamic and extrahypothalamic CRH systems is capable of determining the stress response. Disturbance in the CRH system functioning is associated with the occurrence of addictions, anxiety, post-traumatic stress syndrome, and major depressive disorder. Prevention of such negative consequences is the most important scientific-practical task. The CRH involvement in the formation mechanisms of such a variety of pathologies, reflecting the stress effects on brain functioning, suggests that the extrahypothalamic CRH system may be a potential molecular target for preventing the negative consequences of stress with a high therapeutic potential, because сhronic elevated CRH concentrations have been found in the cerebrospinal fluid of patients with mood disorders, depression, and post-traumatic stress disorder

## Conflict of interest

The authors declare no conflict of interest.
